# Expression of CX3CL1 in the neoplastic lung tissue of squamous cell lung cancer

**DOI:** 10.7150/jca.116713

**Published:** 2025-09-29

**Authors:** Alfonso Salgado-Aguayo, Selma Rivas-Fuentes, Ma. Eugenia Vázquez-Manríquez, Maribel Soto-Nava, César Luna-Rivero, Edgar Sevilla-Reyes, Santiago Ávila-Ríos

**Affiliations:** 1 Laboratory of Research on Rheumatic Diseases, Instituto Nacional de Enfermedades Respiratorias Ismael Cosío Villegas, Mexico City, Mexico.; 2 Laboratory of Transcriptomics and Molecular Immunology, Instituto Nacional de Enfermedades Respiratorias Ismael Cosío Villegas, Mexico City, Mexico.; 3 Department of Pathology. Instituto Nacional de Enfermedades Respiratorias Ismael Cosío Villegas, Mexico City, Mexico.; 4 Center for infectious disease research. Instituto Nacional de Enfermedades Respiratorias Ismael Cosío Villegas, Mexico City, Mexico.

**Keywords:** Squamous cell lung cancer, CX3CL1, CX3CR1

## Abstract

Lung cancer has one of the highest mortality rates. Although epidermoid lung cancer is one of the most prevalent subtypes of lung cancer, no targeted therapy is currently available for this type of cancer. CX3CL1 is a chemokine that has emerged as a potential molecular target for several malignancies. Chemokines direct the migration of various cell types to the tumor and influence tumor cell behavior. To date, little information is available on the role of this chemokine in squamous cell lung cancer. Using immunofluorescence, we evaluated the expression of CX3CL1 in histological specimens of neoplastic lung tissue from squamous cell lung cancer patients. CX3CL1 was expressed in squamous neoplastic lung tissues at all grades of tumor differentiation. We found this chemokine in the cytoplasm and nucleus of non-transformed cells in the adjacent tissue, but it was infrequent in the nucleus of neoplastic cells, which could have biological relevance.

## Introduction

Squamous cell lung cancer is the second most prevalent type of non-small cell lung cancer, with the highest prevalence and mortality rates 1. It mainly affects individuals in their seventh decade of life and is strongly associated with smoking 2. Currently, there is no specific treatment for squamous cell lung cancer. In advanced stages, patients are generally treated with platinum-based chemotherapeutic drugs, which have limited efficacy and various adverse effects. 3. Squamous cell lung cancer originates in flat cells that line the inside of the airways and is commonly found in the center of the lung 2,4.

Chemokines are a family of small soluble molecules that direct cell migration during homeostasis, infection, and/or inflammation 5. In addition, these molecules allow the spatial organization of cells in tissues, induce the release of cytokines, and facilitate biological processes, such as angiogenesis 6. In tumor cells, some chemokines induce proliferation and resistance to cell death, promote angiogenesis, and direct their migration to metastatic sites 7 and help tumor cell establishment in metastatic niches, among other functions 8.

CX3CL1 (or fractalkine) is a chemokine that has recently gained relevance in cancer 9. It is an atypical chemokine because it is synthesized as a membrane-bound protein that functions as an adhesion molecule. Under certain circumstances, the membrane-bound form of CX3CL1 is enzymatically cleaved to release the chemokine domain 10. Soluble CX3CL1 acts as a chemoattractant for cells expressing its receptor CX3CR1, including CD8+ T lymphocytes, NK cells, and patrolling monocytes, among others 9. The role of CX3CL1 may vary significantly between different types of cancer 11. In colon carcinoma, CX3CL1 has been associated with immune response evasion 12, while in lung cancer, there is evidence that CX3CL1 has a dual role, on the one hand favoring the antitumor response through the recruitment of various effector cells 13, and on the other hand, its presence has been related to metastasis and, therefore, with disease progression. Several tumor lung cells also express CX3CL1 receptor, and exogenous stimulation with soluble CX3CL1 induces their migration through mechanisms dependent on the Focal adhesion kinase and Steroid receptor coactivator (FAK-Src) complex 14 and phosphorylation of cortactin, a protein associated with the cytoskeleton 15.

Little is known about CX3CL1 and CX3CR1 in cancer, particularly lung cancer. Some studies in experimental mouse models of carcinogenesis indicate that CX3CR1 deficiency is associated with the presence of larger tumors 16, whereas overexpression of CX3CL1 is associated with smaller tumors, increased infiltration of dendritic and increased survival 13, meaning that CX3CL1 could have an anti-neoplastic effect. On the other hand, there is also evidence indicating that CX3CL1 and its receptor favor tumor cell metastasis and its establishment in a favorable niche through immune response evasion mechanisms related to the recruitment of myeloid suppressor cells and PD-1 12 and formation of metastases 17. In human lung cancer, CX3CL1 is important for the development of bone metastasis 14,18. In patients with lung adenocarcinoma, a high level of CX3CL1 expression in the lungs seems to be related to shorter survival in smokers, only one-third of that found in the non-smoking group; however, no differences were found between the squamous lung cancer groups 19. Therefore, CX3CL1 may have different functions in patients in specific contexts and among different subtypes of lung cancer. Although studies on the role of CX3CL1 in lung cancer in humans are few, some have proposed CX3CL1 as a target molecule for immunotherapy 20. Thus, there is an urgent need to expand basic research data on CX3CL1 in lung cancer, starting with characterization of its expression in human neoplastic lung tissue.

Regarding the expression of CX3CL1 in squamous cell lung cancer, the information available in the literature is also very scarce and is not well documented (it is not possible to observe important details because the images are shown at a very low magnification/resolution) and has not yet been fully explored. Zhou *et al.* 2016 report it but only show the expression of CX3CL1 in adenocarcinoma 21, which generates uncertainty with respect to the reported findings and Su *et al.* 2018 also reported CX3CL1 positivity in an immunohistochemical squamous cell carcinoma tissue 19 sample without assessing the intracellular localization of this molecule. In the present study, we analyzed the expression pattern in squamous cell lung neoplastic tissue using immunofluorescence to evaluate the intracellular localization of CX3CL1. In this study, we analyzed the expression of CX3CL1 by immunofluorescence, in neoplastic lung tissue from patients with epidermoid lung cancer.

## Methods

### Ethic statements and biological samples

The study protocol and the waiver of informed consent, with the use of de-identified archival material, were approved by the institutional Research Ethics Committee (B15-19), in accordance with the Declaration of Helsinki. This retrospective descriptive study included histological samples from seven patients with a confirmed diagnosis of squamous cell lung cancer. The specimens consisted of formalin-fixed, paraffin-embedded remnants of diagnostic or therapeutic lung biopsies (surgical resections). The patients' clinical and histopathological information was collected from their medical records. Inclusion criteria comprised: a histopathological diagnosis of squamous cell lung cancer, availability of sufficient lung tissue for analysis, and accessible clinical records with relevant demographic and comorbidity data. Exclusion criteria included: incomplete clinical or pathological records or available records. 2 samples were excluded.

### Patient comorbidities

Specimen HS-1 was from a patient with chronic obstructive pulmonary disease (COPD) and glaucoma; HS-2 from a patient with type 2 diabetes mellitus; HS-3 and HS-4 from patients with COPD; HS-5 from a patient with hypertension; and HS-6 and HS-7 from patients for whom no comorbidities were recorded.

### Tissue preparation and immunohistochemistry

Histological sections (3 µm) were cut from the remnants of the formalin-fixed, paraffin-embedded biopsies. Subsequently, the tissues were deparaffinised and rehydrated. A few sections from each sample were stained with hematoxylin and eosin. For immunohistochemistry (IHC) and immunofluorescence, antigen recovery was performed using a pressure cooker in citrate buffer. Subsequently, the samples were blocked with a commercial solution of casein (Vector Laboratories; Newark, California, USA), treated with 0.5% triton in PBS for 30 min, and washed with PBS 1X. To determine tumor type, routine IHC was performed using antibodies against a panel of diagnostic markers.

### Immunofluorescence

After antigen retrieval and triton permeabilization, the samples were incubated with goat anti-CX3CL1 (ab193935, Abcam; Cambridge, MA, USA) overnight at 4°C. The next day the cells were washed (0.1% PBS-Tween 20) and treated with Cy3 donkey anti-goat IgG secondary antibody (705-165-147, Jackson ImmunoResearch; Pennsylvania, USA) for 1h at RT. Subsequently, samples were washed, and their autofluorescence was quenched with Vector® TrueVIEW® kit (Vector Laboratories) according to the manufacturer's instructions. The samples were washed again with PBS, and the nuclei were stained with 4.3 µg/ml DAPI solution for 5 min. Finally, all samples were mounted in ProLong Gold antifade mountant (Thermo Fisher Scientific; Massachusetts, USA) and stored at 4°C in the dark. Samples were imaged using an FV-1000 confocal microscope (Olympus; Tokyo, Japan), using a 60X/1.4 NA oil immersion objective. Twin sections treated with blocking buffer instead of primary antibodies were used as the autofluorescence controls. For DAPI, the samples were excited with a 405 nm laser and imaged in the 425-475 nm range, whereas for Cy3, the samples were excited at 559 nm and imaged in the range of 570-625 nm.

## Results

In this brief report, we describe seven lung tissue samples from patients with a confirmed clinical diagnosis of lung cancer, with pathological confirmation of the squamous histological subtype. The combination of p40 positivity (a recent molecular marker with reported 100% specificity and high sensitivity for squamous cell lung carcinoma), and thyroid transcription factor-1 (TTF-1) negativity confirmed the squamous lineage of lung cancer 22. Accurate histopathological classification of lung cancer is relevant for selecting appropriate therapeutic strategies and minimizing adverse outcomes. For example, bevacizumab therapy is known to be associated with bleeding in patients with squamous cell lung cancer 22. Therefore, some cases were tested with the classic markers for squamous cell lung cancer: cytokeratin (CK) 2, 5/6 and 7, and p63. Their histopathological characteristics are summarized in Table 1. In histological specimen 4, focal positivity for TTF-1 was found, i.e., a positive immunoreaction in a small group of cells (+/+++). For pathological classification of this sample, it was necessary to use an additional panel of antibodies, including Napsin, CD56, Synaptophysin, and CK20, which had a negative immunoreaction (table 1). Napsin is positive in lung adenocarcinoma 23, CD56 and Synaptophysin are positive in neuroendocrine tumors 24 and CK20 is negative in squamous cell lung cancer 25. The sample was positive for CK 5/6, CK7, P40, and P63.

**Figure 1:**
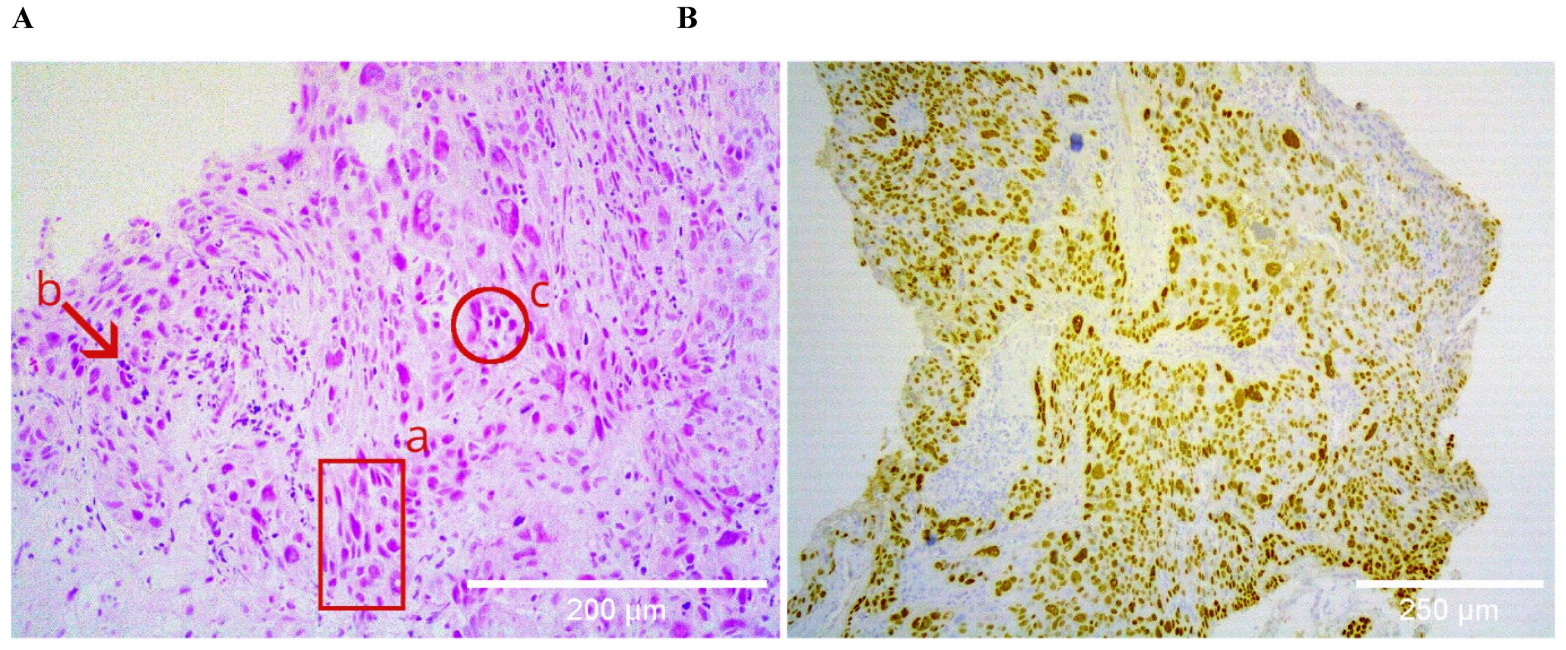
A shows the hematoxylin-eosin staining of a poorly differentiated squamous cell carcinoma sample, at 20X. The bronchial wall is replaced by medium and large neoplastic cells that invade the submucosa; the cells present pleomorphic nuclei (a), are hyperchromatic (b), and form clusters (c). B shows positive nuclear immunoreactive staining for p40 in the same sample, confirming the histological lineage of squamous carcinoma.

Histological specimens included cases with different degrees of histopathological differentiation (Table 2). Figure 1 shows a brightfield micrograph of one of the histological specimens processed with hematoxylin-eosin staining, the cells present pleomorphic nuclei and some of them hyperchromatic (A) and a stain representative of an immunohistochemistry with positive immunoreaction for the p40 antibody (B), where abundant cells positive for this marker are observed.

**Figure 2:**
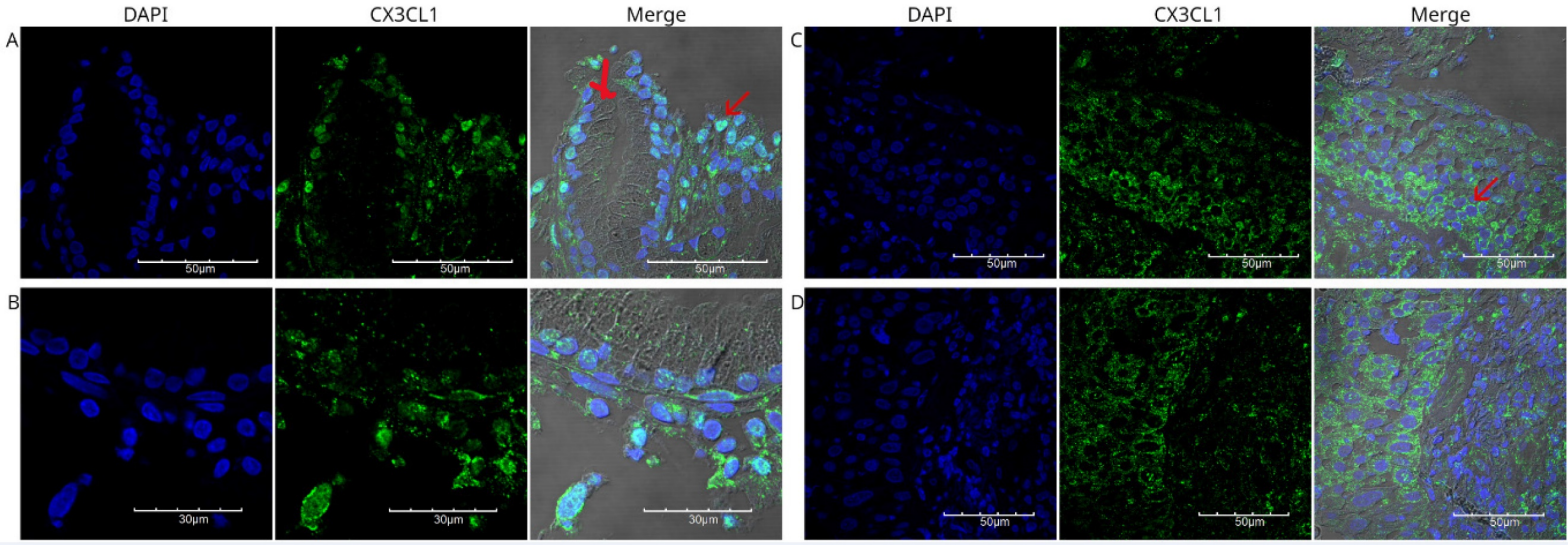
Immunofluorescence detection of CX3CL1 in histological specimen 1, an invasive and moderately differentiated squamous cell carcinoma. Images were acquired using a 60X/1.42 NA objective. In A and B, different fields of bronchial epithelium are shown, surrounded by cells with abundant cytoplasm and pleomorphic nuclei, which are compatible with neoplastic cells with nuclear positivity for CX3CL1 (arrow). C and D show the cellular mass of the tumor tissue, with cells expressing CX3CL1 in the cytoplasm but not in the nucleus. The nuclei were stained with DAPI and are shown in blue, whereas CX3CL1 immunostaining is shown in green. A merged image of DAPI, CX3CL1, and transmitted light is also shown.

We analyzed the expression of CX3CL1 in squamous neoplastic lung tissues with different degrees of pathological differentiation. Figure 2 shows different fields of a histological specimen of moderately differentiated invasive squamous cell carcinoma from the right upper lung lobe. Bronchial epithelium and neoplastic cells can be seen. Neoplastic cells were identified by their pleomorphic nuclei, as evidenced by DAPI staining. The nuclei are shown in blue. CX3CL1 protein expression is shown in green, and the merged image shows an overlay of both markers and transmitted light. In this composite image, the neoplastic cells present abundant cytoplasm and exhibit positive immunoreactivity for CX3CL1.

**Figure 3:**
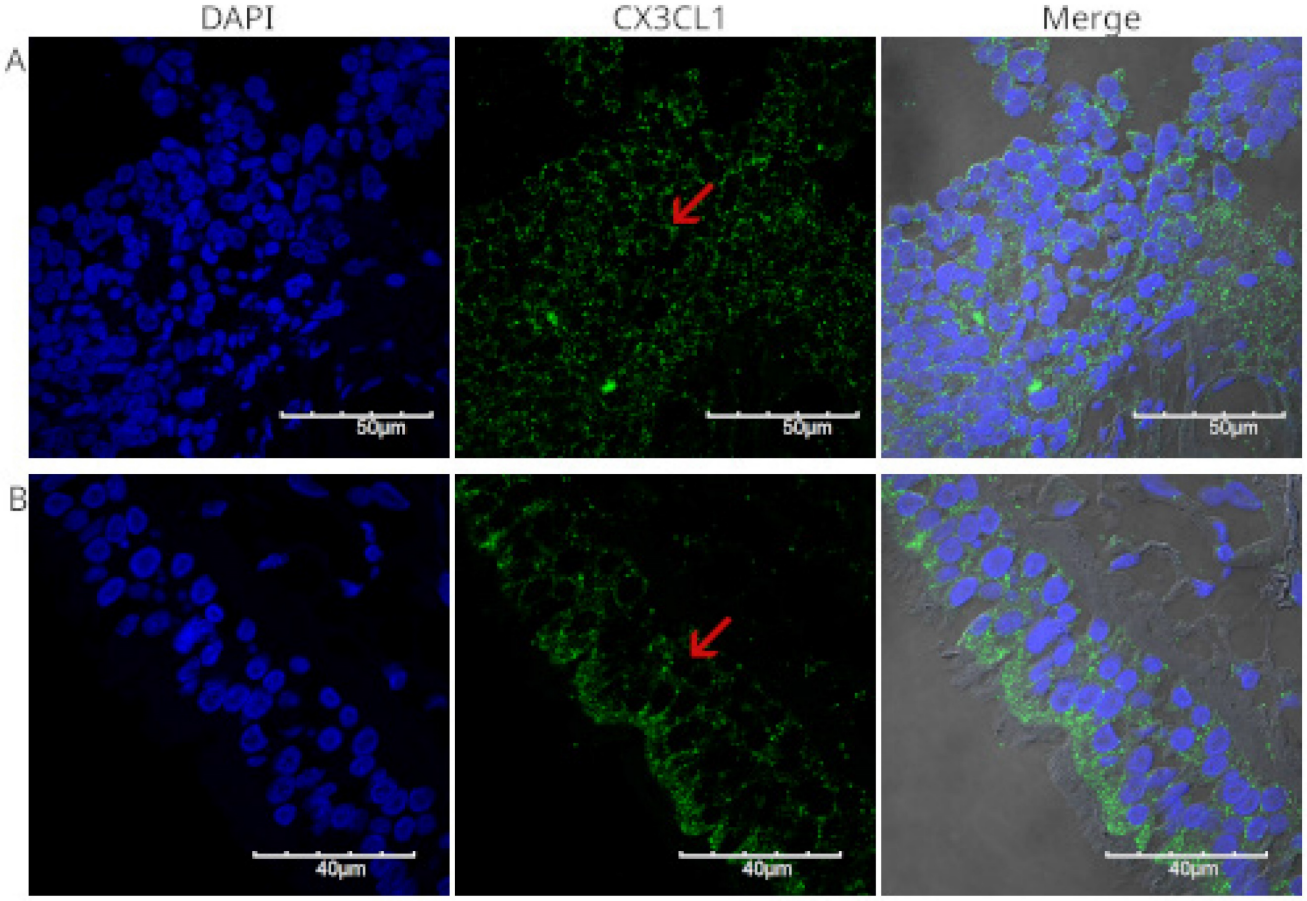
Immunofluorescence detection of CX3CL1 in histological specimen 2, a poorly differentiated squamous cell carcinoma. Images were acquired using a 60X/1.42 NA objective. Panels A and B show fragments of the respiratory mucosa and lung parenchyma, with a neoplasm containing small- and medium-sized cells with scant cytoplasm and large, irregular, and pleomorphic nuclei arranged in nests and mantles. DAPI staining is shown in blue, and CX3CL1 in green. The red arrow indicates a cell without nuclear staining, in which CX3CL1 was observed to be expressed only in the cytoplasm.

**Figure 4:**
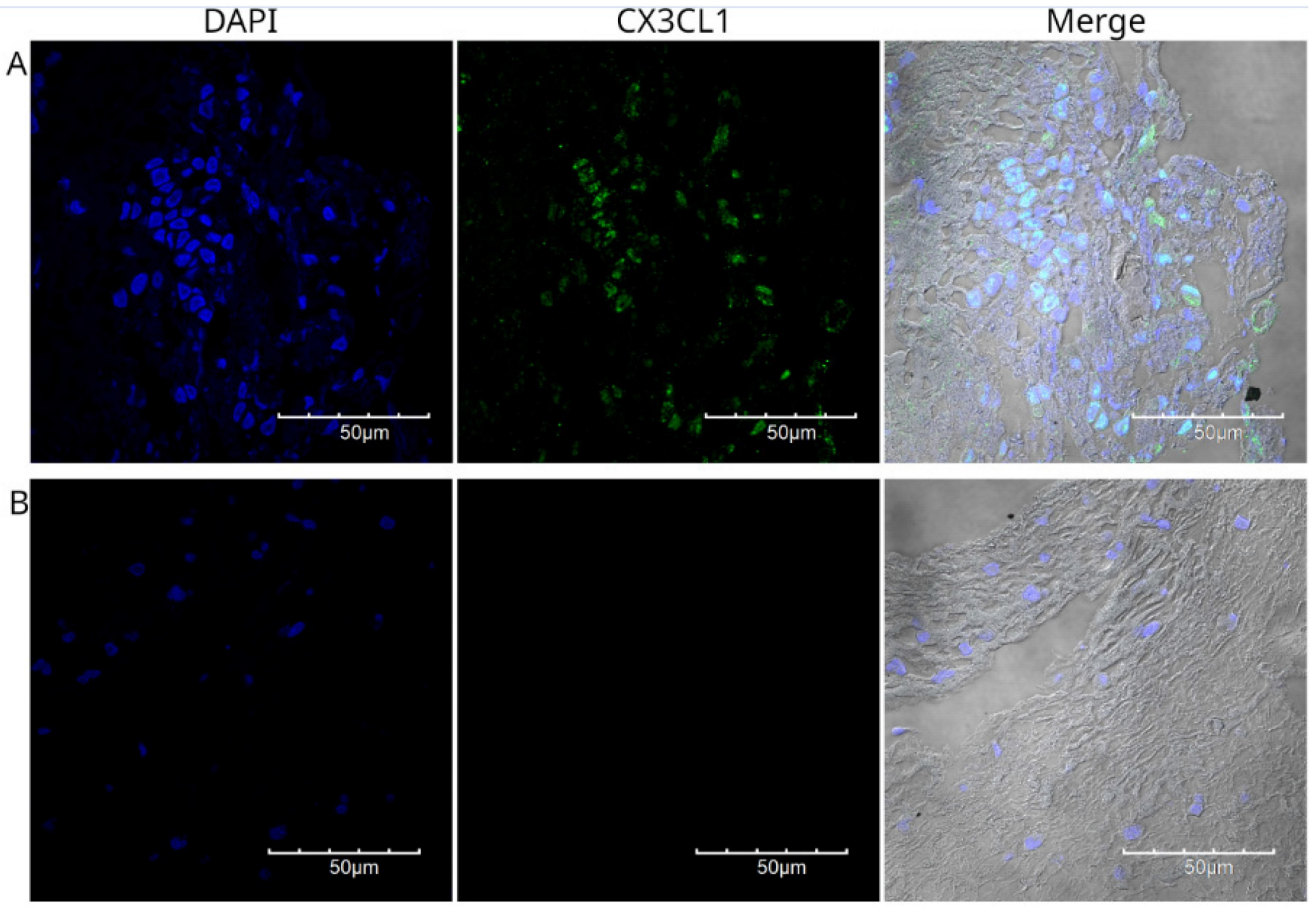
Immunofluorescence detection of CX3CL1 in histological specimen 3, a well-differentiated squamous cell carcinoma. Images were acquired using a 60X/1.42 NA objective. The nuclei were stained with DAPI and are shown in blue. CX3CL1 immunostaining is shown in green. In contrast to the strong staining in the nucleus, the cells showed very low cytoplasmic staining. Panel B shows a no primary control in which the same specimen was incubated with the secondary antibody only.

Figure 3 shows the staining of a poorly differentiated squamous cell carcinoma from the upper lobe of the right lung. Fragments of lung parenchyma were observed in this histological section. The neoplastic cells were small to medium in size, had a scant cytoplasm, presented a nested arrangement, and were positive for CX3CL1. Positive immunoreactivity for CX3CL1 was observed in the cytoplasmic and perinuclear regions. Figure 4 shows a well-differentiated squamous cell carcinoma sample from the lower lobe of the right lung. Panel A shows low-intensity positive staining for CX3CL1. In all analyzed samples, a twin slice was stained with the secondary antibody and no primary antibody to evaluate the signal due to autofluorescence. Figure 4B shows a representative image of the autofluorescence from the same histological specimen.

**Figure 5:**
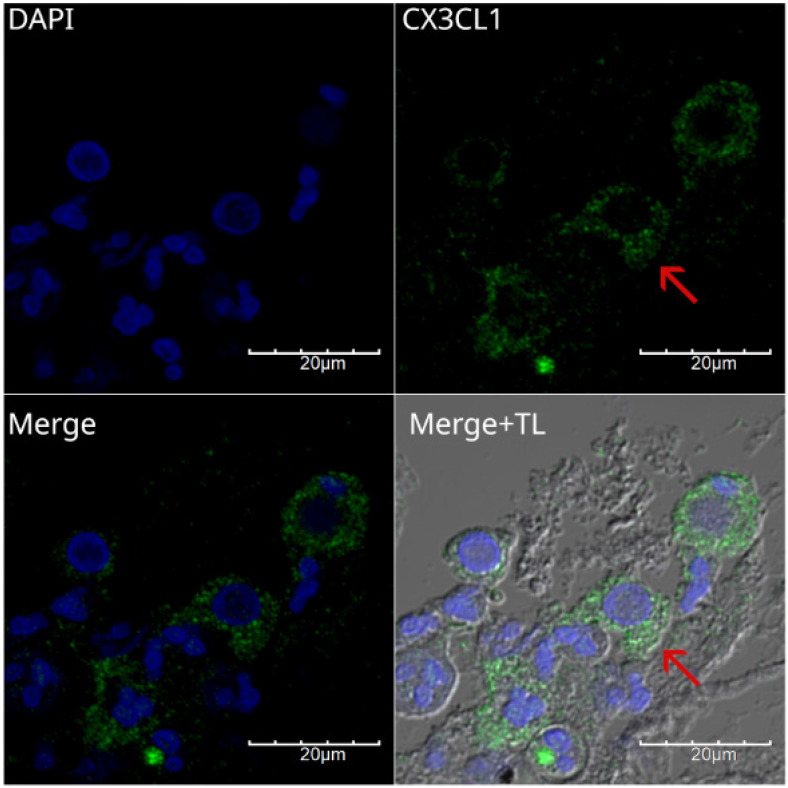
Immunofluorescence for the detection of CX3CL1 in histological specimen 4, a moderately differentiated squamous cell carcinoma. Images were acquired using a 60X/1.42 NA objective. A scan zoom of 3.5 × was applied, resulting in a final field of view of 60 × 60 µM. Nuclei were stained with DAPI and are shown in blue, whereas CX3CL1 immunodetection is shown in green. The lower left panel shows a merged image of DAPI and CX3CL1 without transmitted light to better observe the perinuclear staining pattern, while the lower right panel also includes transmitted light. The red arrow indicates a cell with nuclear expression of CX3CL1. Notably, some multinucleated cells in this sample lacked CX3CL1.

All specimens analyzed for CX3CL1 exhibited perinuclear and cytoplasmic staining. To show this more clearly, Figure 5 presents a higher-magnification micrograph of histological specimen 4, a case of moderately differentiated epidermoid carcinoma obtained by bronchoscopy and brushing. In panel 5B, the composite image shows cells where CX3CL1 is practically absent in the nucleus, contrasting with previous reports, where CX3CL1 was observed in the nucleus in lung tissue of histological specimens of idiopathic pulmonary fibrosis and in normal lung tissue 26. Notably, some multinucleated cells in this sample lacked CX3CL1.

It has also been reported that CX3CL1 is cleaved in its intracellular region by intracellular secretases; once released, it translocates to the nucleus 27. On the other hand, a low tissue signal for CX3CL1 could also suggest an increased generation of soluble CX3CL1 because CX3CL1 also exists as a chemotactic soluble molecule produced by proteolytic cleavage of the extracellular region 10. Both aspects could have important implications for the pathophysiology of squamous cell lung cancer as discussed below.

## Discussion

The pattern of CX3CL1 expression found in squamous cell lung cancer tissues could have important clinicopathological applications and biological implications in the pathophysiology of this type of cancer. Previous studies have reported the expression of CX3CL1 in squamous cell lung cancer tissues by immunohistochemistry 21,19, in which the location of this chemokine cannot be discriminated between the membrane, cytoplasm and nucleus, because in the case of IHC, counterstaining that masks the nuclei are frequently used.

Previously, we demonstrated the presence of CX3CL1 in the nuclei of fibroblasts 26. In the present study, we analyzed the expression pattern of CX3CL1 in squamous cell lung neoplastic tissues: colocalization between DAPI and fluorescent staining of CX3CL1 allowed us to evaluate the nuclear localization of this chemokine. We present cases of squamous cell lung carcinoma of different degrees of pathologic differentiation, where the poorly differentiated cases were properly classified (Table 1), which is important because in cases of poorly differentiated tumors, the differentiation between these two subtypes is complex. The frequent absence of positive immunoreactivity for CX3CL1 in the nucleus of squamous cell lung cancer cells could be part of a differential local transcriptomic fingerprint and may be related to the transformation processes in this type of cancer. This is highly relevant because it has been reported that an intracellular portion of CX3CL1 generated by secretases can translocate to the nucleus and induce gene transcription 27.

We found that the analyzed samples showed a generalized pattern of low-intensity CX3CL1 immunoreactivity. This characteristic could be related to an increase in the generation of soluble CX3CL1, as this chemokine is cleaved by various enzymes such as ADAM10, which are potentially found in the pulmonary microenvironment and could be relevant to the outcome of the disease 28. On the other hand, some histological samples analyzed in this study come from patients with DM2 comorbidities or systemic hypertension. However, we observed a homogeneous expression pattern among the samples analyzed.

In future studies it will be important to analyze whether the pattern of CX3CL1 expression in lung cancer could be a differential marker between squamous cell lung cancer and other sub types of lung cancer. It is also important to clarify whether CX3CL1 could be a potential marker of response in some contexts, such as specific antineoplastic treatment. Drug treatment may also have an impact on the effect of CX3CL1 in neoplasms. For example, in a murine model of humanized breast cancer, the group of mice that received tumor cells with higher expression of CX3CL1 developed larger tumors, but the group that was also treated with Trastuzumab (an anti-HER-2 antibody) had a lower proportion of animals with metastases 20. Therefore, the increased presence of CX3CL1 has a pro-tumorigenic role in untreated animals, but in Trastuzumab-treated animals, overexpression of CX3CL1 appears to inhibit progression.

## Conclusions

CX3CL1 is expressed in neoplastic lung tissue of epidermoid lung cancer, cells positive for this chemokine are observed in the different histopathological stages and in almost all cells. The localization of CX3CL1 in epidermoid lung cancer neoplastic tissue is mainly cytoplasmic and perinuclear and is generally absent in the nucleus.

## Figures and Tables

**Table 1:**
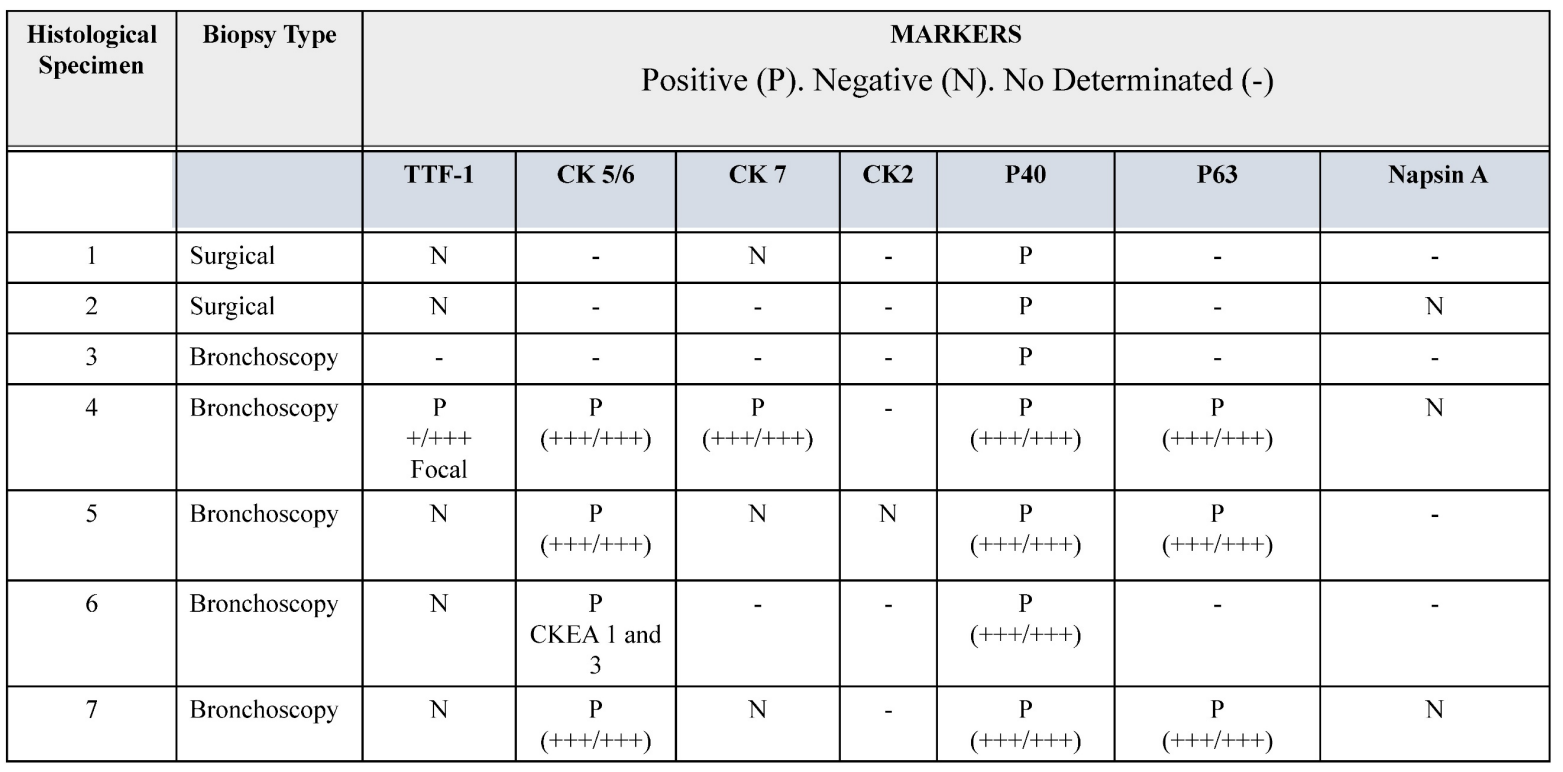
Immunohistochemical features of biopsies

Abbreviations: TTF-1. Thyroid transcription factor 1. CK. Cytokeratin. P65 antibody for p65 gene. P40 antibody for ΔNp63 gene.

**Table 2:**
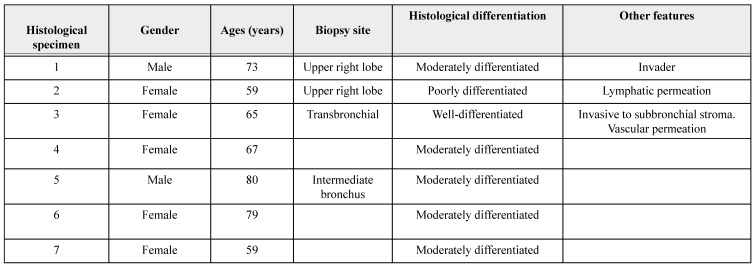
Demographic characteristics of patients and their pathologic diagnosis

## Abbreviations

TTF-1: Thyroid transcription factor 1
CK: Cytokeratin
P65: antibody for p65 gene
P40: antibody for ΔNp63 gene
